# Changes in Feed Proanthocyanidin Profiles during Silage Production and Digestion by Lamb

**DOI:** 10.3390/molecules25245887

**Published:** 2020-12-12

**Authors:** Marion Girard, Annika Lehtimäki, Giuseppe Bee, Frigga Dohme-Meier, Maarit Karonen, Juha-Pekka Salminen

**Affiliations:** 1 Agroscope, Tioleyre 4, 1725 Posieux, Switzerland; marion.girard@agroscope.admin.ch (M.G.); giuseppe.bee@agroscope.admin.ch (G.B.); frigga.dohme-meier@agroscope.admin.ch (F.D.-M.); 2 Natural Chemistry Research Group, Department of Chemistry, University of Turku, Vatselankatu 2, FI-20014 Turku, Finland; annikaleht@outlook.com

**Keywords:** condensed tannins, digestive tract, lambs, selected reaction monitoring, silage, UPLC-MS/MS, wilted plant

## Abstract

Proanthocyanidins are plant specialized metabolites which are beneficial to animal nutrition and health. This study determined how proanthocyanidin profiles of sainfoin (*Onobrychis viciifolia*) and birdsfoot trefoil (*Lotus corniculatus*) change during the forage conservation process and along the digestive tract of lamb. We determined soluble, protein- and fiber-bound proanthocyanidins by spectrophotometric methods and soluble proanthocyanidin profiles by UPLC-MS/MS. During the conservation process, the total proanthocyanidin contents reduced in both forages and the relative proportion of insoluble proanthocyanidins increased, especially in sainfoin. The soluble proanthocyanidins, their mean degree of polymerization and the relative prodelphinidin share declined in both feed species. In the abomasum of lambs fed sainfoin silage, most of the proanthocyanidins were in insoluble form bound to proteins and fibers, but in the small and large intestines, the proportion of soluble proanthocyanidins increased again. For lambs fed birdsfoot trefoil, the trend was not so clear as proanthocyanidins were already mainly soluble in the abomasum. Nevertheless, a large part of soluble proanthocyanidins was recovered in the digestive tract but could not be detected by the UPLC-MS/MS method used. This study suggests that proanthocyanidins have probably been metabolized in the digestive tract by the resident microbiota.

## 1. Introduction

The plant tannins, i.e., proanthocyanidins (PAs, syn. condensed tannins), hydrolysable tannins and phlorotannins, are a unique group of plant specialized metabolites. PAs are the most widely distributed group of tannins and they occur ubiquitously in different plant parts. The PAs are of great interest in human nutrition and medicine, but they also have been shown to benefit animal health and performance [1,2].

**Figure 1:**
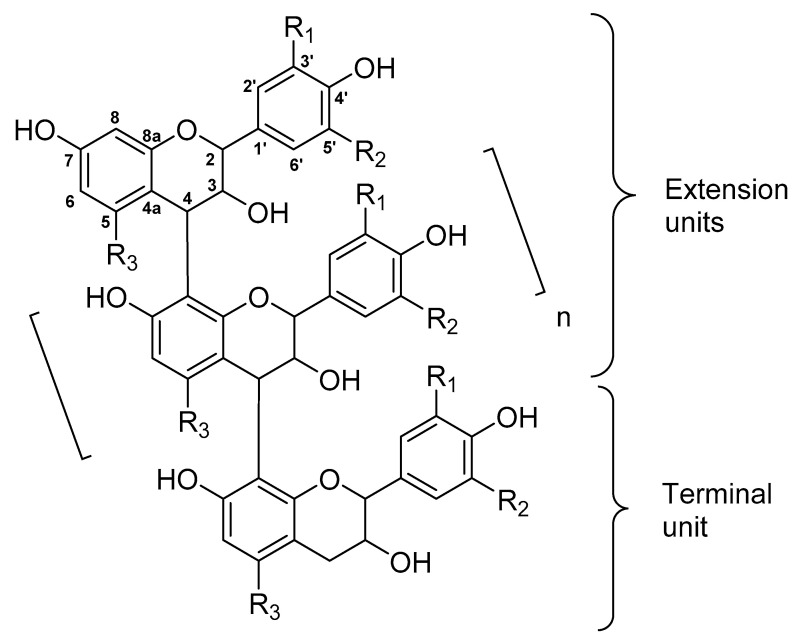
Model structure and numbering of B-type proanthocyanidins with C4→C8 linkages. R1, R2 and R3 can be H, OH or substituted. For procyanidins, R1 = R3 = OH, R2 = H; for prodelphinidins R1 = R2 = R3 = OH.

The PAs are oligo- and polymers consisting of two or more flavan-3-ol monomeric units with different hydroxylation patterns (Figure 1). The most common PAs are procyanidins (PCs) consisting of (+)-catechin or (−)-epicatechin and prodelphinidins (PDs) consisting of (+)-gallocatechin and (−)-epigallocatechin units having 2*R*, 3*S* and 2*R*, 3*R* stereochemistry, respectively. In B-type PAs, the monomers are linked via C4→C6 or C4→C8 bonds. The A-type PAs have an additional C2→O→C7 or C2→O→C5 ether bond. In addition, PAs can be substituted, for example galloylated or glycosylated [3,4,5,6]. The diversity of PAs derives mainly of the above-mentioned hydroxylation patterns, types of interflavanoid linkages, differences in stereochemistry at C2 and C3 and the degree of polymerization (DP). The structural diversity of PAs makes their analysis challenging. However, the nature of the monomeric units (so called PC/PD ratio) and the mean DP (mDP) of soluble PAs can be determined rather easily [5,7,8]. Ultraperformance liquid chromatography‒diode array‒tandem mass spectrometry (UPLC-MS/MS) is the most efficient tool. The consecutive selected reaction monitoring allows the rapid determination of the total PA, PC and PD contents and PC/PD ratio, in addition to the estimation of mDP and how the different PCs and PDs are distributed along the chromatographic PA hump [7].

In nature, PAs are present in soluble and insoluble forms, for example over 90% of PAs in watermelon pilea leaves (*Pilea cadierei*) and in Turkey oak heartwood (*Quercus cerris*), and over 75% of PAs in pine bark (*Pinus sylvestris*) are in insoluble form [9,10,11]. The PAs can bind with proteins and fibers. The PAs with a high number of hydroxyl groups (hydrogen bonds) or aryl groups (hydrophobic interactions) or a high mean degree of polymerization (mDP, more binding sites) bind stronger to proteins [12,13]. In addition to the molecular size, the stereochemistry of the monomeric flavan-3-ol units may affect the efficacy of binding: the binding of (+)-catechin to proteins is stronger than that of (−)-epicatechin [14,15]. The increase in the rate of galloylation of PAs also increases their binding efficacy [16].

The analysis of insoluble PAs is more challenging than that of soluble PAs, since the methods for insoluble PAs cannot analyze the native PAs, but only their degradation products. The most often used method is BuOH/HCl depolymerization, which was initially developed for soluble PAs by Swain and Hillis [17] and later improved by Bate-Smith [18] and Porter et al. [19]. The method has also been used for insoluble PAs [20]. Recently, Grabber et al. [21] enhanced the traditional BuOH/HCl method by using acetone as cosolvent with BuOH/HCl (later called BuOH/HCl/Ace method) and thereby increasing the anthocyanidin yields, especially when used directly on the plant material. Another common method for the analysis of insoluble PAs is their acid catalyzed depolymerization in the presence of nucleophile, i.e., thiolysis using benzyl mercaptan as a nucleophile or pholoroglucinol degradation with pholoroglucinol [22,23]. Reaction products can then be analyzed by LC-MS. In addition, these methods were initially developed for soluble PAs, but have been later utilized for insoluble ones [24,25,26,27]. Nevertheless, the traditional thiolysis might underestimate the PA contents in old plant tissues, such as outer bark, or in silages [28,29].

For several years, PAs have proven to be beneficial for livestock, especially for ruminants. Numerous studies reported improved growth performances, fertility, milk and wool production and reduced methane emissions and parasitic burden in ruminant fed PA-rich forages [2,30]. More recently, PAs have been shown to positively affect product quality by increasing the content of favorable fatty acids, such as *n*-3 fatty acids, and by decreasing unpleasant flavors present in animal grazing pasture [31]. For example, feeding of sainfoin increased *n*-3 fatty acid levels in milk, Gruyère-type cheese and lamb meat and reduced skatole content in the perirenal fat of lamb [32,33]. However, owing to the season and the feeding system, forages can be either grazed (fresh) or offered as conserved feed in the form of hay, pellets or silage. It is now known that PA composition and content in forage legumes change during wilting [34]. In fact, a remarkable effect on the PA solubility has been observed between the different conservation processes; it has been shown that soluble PAs make 74% of total PAs in fresh sainfoin material, but only 52% in pelleted sainfoin and merely 15% in ensiled sainfoin [2]. Therefore, it is important to understand how the PA composition and bioactivity change during processing and along the digestive tract to effectively improve animal performance and health.

In this study, we wanted to examine in detail: (1) What is the soluble vs. insoluble PA composition in two temperate legume forages: sainfoin (*Onobrychis viciifolia*) and birdsfoot trefoil (*Lotus corniculatus*)? (2) How does the PA composition change during the feed processing from fresh feed to silage? (3) How does the PA composition change along the digestive tract of sheep? We used sainfoin and birdsfoot trefoil as fresh material, wilted material and silage for the studies on PA composition and contents during the feed production. Ensiled materials were used in animal feeding experiments for the studies on PA composition and contents along the digestive tract of lambs. We determined the soluble and insoluble PAs by the well-established Terrill’s method [35] and by the more recent BuOH/HCl/Ace assay [21], and UPLC-MS/MS method [7].

## 2. Results and Discussion

### 2.1. Soluble and Insoluble PAs by BuOH/HCl/Ace Assay

**Figure 2:**
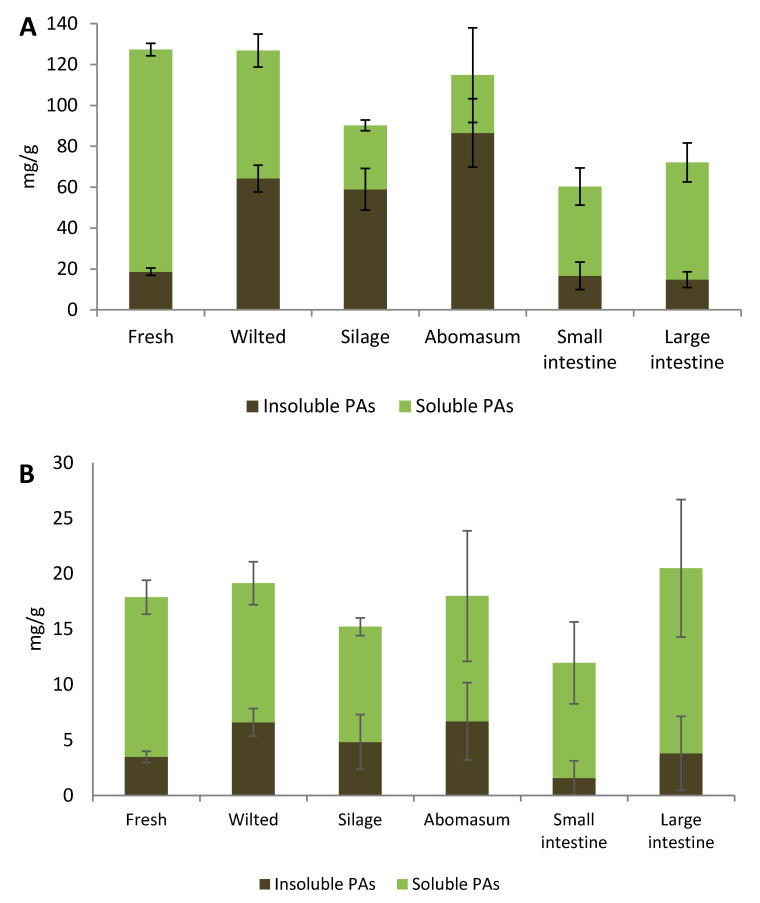
The contents (mg/g dry matter) of soluble and insoluble proanthocyanidins (PAs) in fresh, wilted and silage feed samples of the first harvest and in abomasum, small and large intestines of sheep fed with silage for (**A**) sainfoin (*Onobrychis viciifolia*) and (**B**) birdsfoot trefoil polom (*Lotus corniculatus*) obtained by BuOH/HCl/Ace assay [21].

In all samples, PAs were only detected in the water-soluble fractions and not in the fat-soluble fractions (data not shown). The contents of soluble and insoluble PAs in the different samples are presented in Figure 2. Sainfoin had on average six-fold greater PA contents in fresh, wilted and silage materials than birdsfoot trefoil, which is in agreement with previous experiments [36,37]. During the conservation process of sainfoin, the total PA content (soluble and insoluble) was reduced by 29% in the silage compared with fresh and wilted samples. However, the total PA content remained constant around 17 mg/g DM for birdsfoot trefoil during the forage conservation. This hinted that the PA structures in these two feed materials might be different. Regardless of the total PA content, the forage conservation affected also the PA composition. Forage conservation increased insoluble PA contents while concomitantly decreasing the soluble PA content. This observation was even more noticeable when the ratio soluble:insoluble was expressed. The soluble:insoluble ratio was 85:15, 49:51 and 35:65 for sainfoin and 80:20, 65:35 and 68:32 for birdsfoot trefoil in fresh, wilted and silage respectively. This supported previous findings with similar types of samples [37,38]. The increase in the insoluble fraction may be related to cell damages due the conservation process. Soluble PAs, initially stored in the vacuole, are released in the cytosol where they can react with protein and fibers [39]. Compared with silages, the PA content in the abomasum of lambs fed sainfoin and birdsfoot trefoil was slightly greater. This might be related to a concentration of PAs due to the absorption of nutrients in the rumen.

In the digesta samples, PAs from lambs fed sainfoin were mainly in an insoluble form in the abomasum, counting for 75% of total PAs, whereas 72 and 80% of total PAs were in a soluble form in the small and large intestine, respectively. For lambs fed birdsfoot trefoil, 63%, 87% and 81% of PAs in the abomasum, small intestine and large intestine, respectively, were in a soluble form. A previous study using the Terrill’s method reported greater proportions of insoluble PAs in the digesta of lambs fed *Lotus pedunculatus*: 78%, 96% and 98% in the abomasum, ileum and feces, respectively [40].

As soluble PAs decreased during the forage conservation, we were interested in the better characterization of these soluble PAs using the UPLC-MS/MS. Contrary to insoluble PA fractions, soluble PAs can be characterized with the consecutive selected reaction monitoring methods developed by Engström et al. [7].

### 2.2. The Compositions of Soluble PAs by UPLC-MS/MS

**Figure 3:**
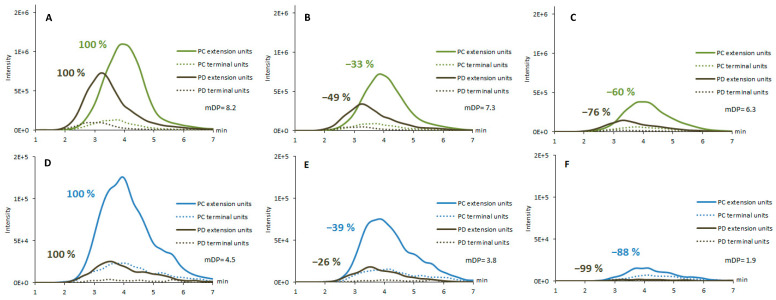
UPLC-MS/MS fingerprints for procyanidin (PC) and prodelphinidin (PD) extension (solid lines) and terminal units (dotted lines) in sainfoin (*Onobrychis viciifolia*) (**A**) fresh feed of the first harvest, (**B**) wilted feed of the first harvest, and (**C**) silage and in birdsfoot trefoil polom (*Lotus corniculatus*) (**D**) fresh feed of the first harvest, (**E**) wilted feed of the first harvest, and (**F**) silage. The fingerprints were obtained by selected reaction monitoring with the following transitions: PC extension unit: *m/z* 287 → 125, PC terminal unit: *m/z* 289 → 245, PD extension unit: *m/z* 303 → 125 and PD terminal unit: *m/z* 305 → 125 [7]. mDP refers to mean degree of polymerization of proanthocyanidins.

The soluble PAs were analyzed by the UPLC-MS/MS method which is capable to detect the PA fingerprints and quantify PC and PD concentrations including the PC/PD ratio and define the mDP [7]. This UPLC-MS/MS method allows the selective detection of PCs and PDs by performing the depolymerization of PAs in the ion source in order to obtain first generation product ions corresponding for the extension and terminal units of PAs. These characteristic ions are formed via quinone methide cleavage and exhibit *m/z* 287 and 289 for PC extension and terminal units, respectively, and *m/z* 303 and 305 for PD extension and terminal units, respectively. These formed depolymerization products are then further fragmented in the collision cell to obtain characteristic second generation product ions which confirm the detection of PAs and enable their quantitative analysis by selected reaction monitoring (SRM) [7]. The method enables the quantification of total PAs, PCs and PDs, in addition to the determination of PC/PD ratio and mDP of PAs. The SRM chromatographic profiles, called PA fingerprints, show how the different PCs and PDs are distributed along the chromatographic hump that is typically obtained for PAs by reversed-phase chromatography [7]. The UPLC-MS/MS fingerprints showed that the PA structures are different in sainfoin and birdsfoot trefoil and the mDP of PAs was in general higher in sainfoin than in birdsfoot trefoil (Figure 3). Differences can be seen between the fresh feed, wilted feed and silage between the species.

As previously observed with the BuOH/HCl/Ace assay, the content of soluble PAs decreased during the forage conservation (Figure 2). This can be seen by the decrease in the fingerprints of both PC and PD extension and terminal units in both feeds. In sainfoin, PC extension units decreased by 33% in wilted and by 60% in silage samples compared with fresh sample. Similarly, a reduction of 49% and 76% of the PD extension units was observed in the wilted and silage samples compared with the fresh sample. Birdsfoot trefoil followed the same trend as sainfoin regarding the modification of the PA fingerprint during the forage conservation. For birdsfoot trefoil, compared with fresh sample, there was a decrease of 39 and 88% for PC extension units and of 26 and 99% for the PD extension units in wilted and silage samples, respectively. Furthermore, the PC/PD ratios changed during the forage conservation. The PC/PD ratio was 67:33 for fresh sainfoin, 73:27 for wilted and 77:23 for silage, and respectively, 87:13 for fresh birdsfoot trefoil, 88:12 for wilted and 99:1 for silage. Accordingly, the PD extension units decreased to a greater extent than PC extension units did and the relative amount of soluble PC increased. In addition, the forage conservation affected the mDP of PAs. The mDP declined from 8.2 to 7.3 and 6.3 in sainfoin and from 4.5 to 3.8 and 1.9 for birsdfoot trefoil in fresh, wilted and silage samples, respectively. All these observations, i.e., the decrease in mDP together with PC and PD concentrations and changes in PC/PD ratio, implied that the largest PAs were the most reactive ones and that PD units were more reactive than PC units. This has been previously demonstrated also by Ropiak et al. [41]. Several hypotheses can be drawn from these observed changes: (i) during forage processing, large polymers are cleaved into smaller ones; (ii) large polymers are oxidized and therefore cannot be detected anymore with this UPLC-MS/MS method; (iii) large polymers are reacting with other compounds such as feed proteins and fibers and are therefore in the insoluble fraction. The latter assumption would be in agreement with the increase in insoluble PA content previously determined with BuOH/HCl/Ace assay and a combination of the aforementioned hypothesis might occur. Regardless forage conservation, the PA fingerprints showed that the PA compositions in sainfoin and birdsfoot trefoil were very different: Therefore, depending on whether animals are fed sainfoin or birdsfoot trefoil, the PA bioactivity may be changed, which might affect animal responses. Indeed, the silage of birdsfoot trefoil contained, in soluble form, mainly PCs (99%), while the sainfoin silage also contained PDs (23%). The mDP of PAs in sainfoin silage (6.3) was also considerably higher than that of the birdsfoot trefoil silage (1.9). Different conservation additives and methods may also affect the composition of PAs differently.

The soluble PA fraction of the digesta samples (abomasum, small and large intestines) were also analyzed by UPLC-MS/MS. However, the fingerprints were really faint, i.e., few soluble PCs and PDs were detected. As soluble PAs were detected in digesta with the BuOH/HCl/Ace assay (Figure 2), the low recovery of soluble PAs by UPLC-MS/MS implies that in the digestive tract, they were further chemically transformed and could not be detected with the PC and PD specific MS/MS method used. After the analysis of soluble PAs, we wanted to investigate the insoluble PAs further by Terrill’s method [35] in order to access and quantify the protein- and fiber-bound PAs.

### 2.3. Soluble, Protein-Bound and Fiber-Bound PAs by Terrill’s Method

**Figure 4:**
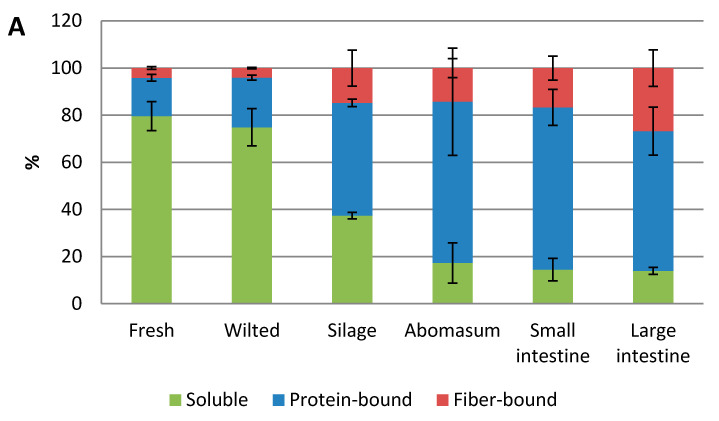

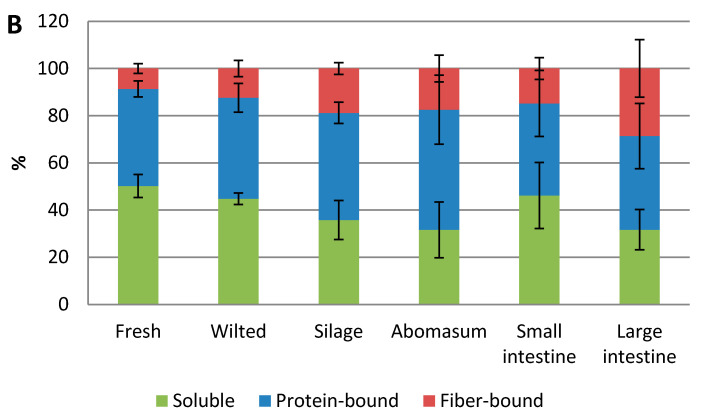
The proportions of soluble, protein-bound and fiber-bound PAs in fresh, wilted and silage feed samples and in abomasum, small and large intestines of sheep fed with silage for (**A**) sainfoin (*Onobrychis viciifolia*) and (**B**) Birdsfoot trefoil polom (*Lotus corniculatus*) obtained by Terrill’s method [35].

The feed materials and digesta samples were extracted and analyzed by Terrill’s method [35] in order to study in more detail the insoluble protein- and fiber-bound PAs. The proportions of soluble, protein-bound and fiber-bound PAs are presented in Figure 4. In general, the actual amounts of soluble and insoluble PAs were lower than the ones obtained by our own extraction method and the BuOH/HCl method of Grabber et al. [21]. This is understandable as the extraction solvent was different. In addition, it has been demonstrated that acetone used in the BuOH/HCl/Ace method of Grabber et al. [21] enhances the anthocyanidin yields by up to 3.2-fold in the PA analysis.

Fiber- and protein-bound PAs were detected in all samples. Most of the insoluble PAs were protein-bound: 80% and 83% in fresh materials, 84% and 78% in wilted materials, 76% and 71% in silages, 83% and 74% in abomasum, 80% and 72% in small intestine and 69% and 58% in large intestine, in sainfoin and birsdsfoot trefoil, respectively. Moreover, during forage conservation, the proportion of fiber-bound PAs increased from fresh material to silage. This increase seemed to be correlated with the 5 %-unit greater NDF content in silage compared with fresh material for both type of plant. The bound PAs increased from silage to abomasum as shown in Figure 2. From Figure 4, we can see that much of that increase was governed by the increase in protein-bound PAs. For sainfoin, the most of PAs in the abomasum and small intestine were protein-bound and their amount decreased in the large intestine. For birdsfoot trefoil, the amount of protein-bound PAs was the greatest in abomasum, but decreases a little in the small or large intestines. For both feed species, the proportions of fiber-bound PAs increased in large intestines. As the large intestine contains a high proportion of fibers, the likelihood that PAs bind fibers in the large intestine increases.

### 2.4. Unidentifiable Role of Insoluble PAs

Plants may contain a significant amount of various insoluble polyphenols, which may have significant biological effects on humans and animals. However, insoluble polyphenols have been studied relatively little, and their analysis are challenging due to their limited extractability, large size and structural diversity [42,43]. The most widely used methods for analyzing insoluble PAs are their depolymerization with BuOH/HCl and various thiolytic methods. However, in these methods the polymeric compounds are degraded prior to more detailed analysis, thereby losing the knowledge of the structure of the initial compounds. Overall, the analysis of insoluble polyphenols has not yet reached the same level as for soluble compounds. The choice of the analytical method, reaction conditions, and standard will have a significant impact on the outcome, making it difficult to compare the results obtained so far, as also seen in this study when comparing Figure 2 and Figure 4. There is also no systematic method for determining all soluble and insoluble polyphenols directly from plant material.

Our work showed the change in the concentrations of soluble and insoluble PAs in sainfoin and birdsfoot trefoil during the forage conservation and in the digestive tract of sheep fed on silage. Our results confirmed that the composition of PAs changed during the forage conservation. The amount of soluble PAs decreased in sainfoin and the amount of insoluble PAs bound to proteins and fibers increased. The amount of soluble PAs in birdsfoot trefoil decreased, while the binding of PAs to feed fiber increased. In addition, along the different steps of the silage production of silage processing, the PC/PD ratio and the mDP value also changed. The mDP value of soluble PAs decreased, which may indicate that especially larger PAs are those that react and bind to proteins. The higher molecular size of PAs is known increase its interaction and binding with proteins [41,42]. It is worthwhile to mention that the composition of PAs, given by the PC/PD ratio and the mDP, in the soluble and insoluble fractions, is different during forage conservation. The PD accounted for 70% of total PAs (soluble and insoluble) in the same fresh and wilted sainfoin when measured with a thiolysis method while in the present study, the PD represented 33 and 27%, respectively, of the soluble PAs, the rest being PC [34]. In addition, the mDPs were 11.5 and 9.2 for total PAs in fresh and wilted sainfoin, respectively, with the thiolysis method against 8.2 and 7.3 in the present study [34]. This combination of results probably highlights that the PD and the larger polymers are mainly present in the insoluble fraction. Therefore, this fraction should not be neglected because PAs bound to feed proteins may protect them from ruminal degradation and might release them in the intestine, as suggested previously by Kariuki and Norton [44].

Understanding the fate of PAs in the digestive tract is challenging. Several studies reported that PAs in sheep are not absorbed as none or few PAs or their metabolites have been detected in the plasma, liver or in urine of sheep fed PA-rich diets [45,46]. However, a clear disappearance of PAs in the feces has been mentioned. A disappearance of 87 % in feces of lambs fed sainfoin pellets was reported in a previous study [47]. In the present study, lambs were solely fed sainfoin or birdsfoot trefoil silage for more than 4 months. By using the same estimated dry matter digestibility of 58% as did Quidaja et al. [47], a PA content of 156 and 26 g/kg DM would be expected in the feces of lambs fed sainfoin and birdsfoot trefoil respectively. Nevertheless, 46% and 78% of total PAs were recovered in the large intestine of lambs fed sainfoin and birsdfoot trefoil with the BuOH/HCl/Ace method. As hypothesized by other authors [47,48], this disappearance may be explained by: (i) a difficulty to extract insoluble PAs, even with thiolytic methods, that bind covalently to other compounds as shown in the present study by the increase in protein- and fiber-bound PAs in the digestive tract and/or (ii) a modification of the initial PAs due to prevailing physio-chemical conditions in the digestive tract and to the ruminal and intestinal microbiota as shown by the inability to detect soluble PAs by UPLC-MS/MS despite a great amount of soluble PAs quantified by BuOH/HCl/Ace method. The previous study of Desrues et al. [48] reported a decrease in mDP of PAs in the digestive tract of calves fed sainfoin pellets, which might strengthen the hypothesis of a degradation of PAs along the digestive tract. In humans, PAs are fermented in the colon to produce metabolites such as phenolic and hippuric acids and valerolactones, that are later absorbed [49]. Therefore, similar mechanisms could occur in the rumen and in the intestine of ruminants and deserve further investigations.

Another interesting point of this study is that a large part of PAs is in a soluble form in the intestine, with the BuOH/HCl/Ace method, supporting the belief of a release of bound PAs in the small intestine. This assertion is even more noticeable for sainfoin where 65% of PAs were bound in silage against 28% in the small intestine. Once again, this pointed out the role of bound PAs. Further studies determining whether the PA composition, such as mDP and PC/PD, and PA form (soluble and insoluble) affect microbial fermentation and metabolite production would help to better understand the fate and the effect of PAs and their metabolites on the microbiome in ruminant.

## 3. Materials and Methods

### 3.1. Preparation of the Experimental Forages

Two PA-containing legumes were sown in March 2012: Sainfoin (*Onobrychis viciifolia*, Perly cultivar) and birdsfoot trefoil (*Lotus corniculatus*, Polom cultivar) without application of mineral fertilizer. Legumes were harvested in July 2012 at early flowering stage for birdsfoot trefoil and full flowering stage for sainfoin. Immediately after cutting, fresh samples were randomly collected and the rest of the harvest was wilted for 24 h on the field before taking wilted samples. One day after cutting, wilted legumes were ensiled without additives in wrapped silage bales and stored for a minimum of 5 months before use. The fresh, wilted and silage samples were all separated into two subsamples. One subsample was used to determine the dry matter (DM) content and the second was stored at −20 °C for further laboratory analysis.

### 3.2. Experimental Lambs and Treatments

The feeding experiment was approved by the Swiss cantonal veterinary office (approval number: 2012_48_FR) and was in compliance with Swiss guidelines for animal welfare. This experiment was a part of a larger trial on growth performances and meat quality [33].During the whole experiment, animals were housed per treatment group in pens of 23 m^2^ and had a free access to fresh water, NaCl licking block and a mineral salt mix (UFA 998; UFA AG, Sursee, Switzerland). Ten White Alpine ram lambs, aged of 53 ± 12 days (mean ± SD) and weighing on average 22 ± 3 kg (mean ± SD), were randomly allocated to one of the two treatment groups balanced for initial body weight. The two groups of five lambs each were either fed the sainfoin silage or the birdsfoot trefoil silage for 123 ± 13 days (mean ± SD). At an average weight of 30 ± 3 kg (mean ± SD), lambs were slaughtered and the contents of their abomasum, small intestine and large intestine were totally collected and frozen at −20 °C for further analysis.

### 3.3. Nutrient Analysis of the Feed

The nutrient content was determined according to standard methods. For the DM concentration, the fresh, wilted and silage samples were dried at 60 °C for 24 h and then at 105 °C for 3 h. To access the chemical composition, fresh, wilted, silage and digesta samples were freeze-dried (Christ Delta 1–24 LSC, Osterode, Germany) and ground to pass a 1-mm sieve (Brabender mill, Brabender, Duisburg, Germany). The DM of the freeze-dried samples was measured gravimetrically after drying at 105 °C for 3h (LECO TGA 601; Mönchengladbach, Germany). A subsequent heating for 4 h at 550 °C allowed the determination of the ash content in order to calculate the organic matter content. Crude protein content was calculated as *n* × 6.25, where *n* was measured according to the Dumas method (AOAC, 968.06) The neutral (NDF) and acid detergent fiber (ADF) were analyzed following standard protocols (AOAC, 2002.4 and 973.18 respectively) using an ANKOM 200⁄220 Fiber Analyzer (Ankom Technology Corporation, Fairport, NY, USA) where NDF was assayed with heat-stable amylase and sodium sulphite. Results of NDF and ADF contents were expressed without residual ash after incineration at 500 °C for 1 h. The general chemical and nutrient compositions, namely DM, OM, CP, NDF and ADF, of the forages used are presented in Table 1.

### 3.4. Extraction of Feed Materials and Digesta Samples for BuOH/HCl/Ace Assays

The freeze-dried materials were ground into fine powder and 100 mg of each was weighted for the extraction. The samples were extracted with three replicates three times with acetone/water (4:1, *v/v*). The extraction volume was 7 mL and the extraction time 1.5 h. At the end, the extraction residue was extracted once with acetone and twice with dichloromethane/methanol (1:1, *v/v*). The extracts and extraction residues were concentrated under nitrogen flow, freeze-dried and weighted. The extract was divided into water- and fat-soluble fractions by liquid–liquid extractions with water/hexane (1:1, *v/v*). The fractions obtained were concentrated by Eppendorf concentrator, freeze-dried and weighed. The water-soluble compounds were dissolved into 2 mL of water and the fat-soluble ones into 500 µL of hexane.

### 3.5. Extraction of Feed Materials and Digesta Samples According to Terrill’s Method 

First, three replicates of 20 mg of each feed or digesta material were extracted with 800 µL of acetone/water (7:3, *v/v*, including 0.1 % ascorbic acid) and 400 µL of diethylether three times according to the method of Terrill et al. [35]. The extraction time was 2 h. The extracts and extraction residues were concentrated, freeze-dried and weighted. Finally, the extracts were dissolved into 1 mL of water.

### 3.6. Determination of Contents of Soluble and in-Soluble PAs in the Feed and Digesta Samples by BuOH/HCl/Ace Assay

BuOH/HCl/Ace followed the procedure by Grabber et al. [21]. Fresh reagent was prepared daily: 40 mg of ammonium iron(III) sulfate (12H_2_O) was added to 3.3 mL of water and 5 mL of 12 M HCl. The solution was mixed for 30 min by a magnetic stirrer and after that, 42 mL of butanol and 50 mL of acetone were added. The aliquots of 50 µL of the water- soluble extract with five replicates were freeze-dried and the corresponding aliquots of fat-soluble extract were evaporated into dryness in the fume hood. Then, 1000 µL of the reagent was added to each sample. The mixtures were vortexed for 5 min and incubated in an oven at 70 °C for 3 h after which they were let to cool down at room temperature for 1 h. The colorimetric analyses were performed as follows: The aliquots of 200 µL were pipetted to a well-plate and the absorbance was read at 550 nm (VWR, Microplate 96 Well PP Flat). The concentrations of PAs were quantified against purified standard of condensed tannins with a mDP of 4.9 and PC:PD ratio of 99:1. The standard was isolated and purified by Sephadex LH-20 column chromatography from the leaves of *Vaccinium vitis-idaea*. The extraction residues and the initial plant and digesta materials were analyzed by weighting first five replicate samples of 2 mg. Then, the 1000 µL of the reagent was added to each sample. The mixtures were vortexed for 5 min and incubated in an oven at 70 °C for 3 h after which they were let to cool down at room temperature for 1 h and finally centrifuged (10 min, 14 000 rpm). The colorimetric analyses of the supernatants were performed as described above.

### 3.7. Determination of Contents of Soluble and Insoluble PAs in the Feed and Digesta Samples by Terrill’s Method

The method followed the procedure by Terrill et al. [35]. Fresh reagents for BuOH/HCl and SDS solution were prepared daily: BuOH/HCl reagent contained 5% of HCl in 2-butanol and SDS solution 10 g/L of SDS and 50 g/L of mercaptoethanol in 10 mM tris/hydrochloride adjusted to pH 8 by NaOH [35]. Before the actual analyses, insoluble PAs in the extraction residues were divided into protein- and fiber-bound. Aliquots of 600 µL of SDS solution were added to the extraction residues after which they were vortexed for 5 min and then incubated in a boiling water bath (98–100 °C) for 45 min. Then, they were let to cool down to the room temperature for 1 h and centrifuged. The supernatants were decanted and the extraction was repeated. The extracts contained the protein-bound PAs and the extraction residues the fiber-bound PAs.

The initial extracts and the extracts containing protein-bound PAs were analyzed by taking 40 µL of the extract and adding 240 µL of BuOH/HCl reagent. The samples were vortexed for 5 min and incubated in a water bath at 95 °C for 3 h. After that, they were let to cool down to the room temperature for 1 h and the colorimetric analyses were performed as described above.

The extraction residues were analyzed by adding 1200 µL of BuOH/HCl reagent and 120 µL of SDS solution to them. The samples were vortexed for 5 min and incubated in a boiling water bath (98–100 °C) for 3 h. The samples were let to cool down to the room temperature for 1 h and after that, they were centrifuged and the colorimetric analyses of the supernatants were performed as described above.

### 3.8. UPLC-MS/MS Analysis

The method followed the procedure by Engström et al. [7]. The water-soluble plant extracts were freeze-dried and dissolved into 300 µL of water. The replicates of water-soluble digesta extracts were combined and freeze-dried. The sample of abomasum was dissolved in 300 µL of water and the samples of small and large intestines into 1000 µL of water. All samples were filtered by 0.2 µm PTFE filters prior the UPLC-MS/MS analysis. The samples were analyzed by UPLC-DAD-ESI-MS/MS instrument (Acquity UPLC^®^, PDA Detector, Sample Manager, Binary Solvent Manager, Xevo TQ; Waters Corp., Milford, MA, USA) using a phenyl column (Acquity UPLC^®^, BEH Phenyl Column, 100 mm × 2.1 mm, 1.7 μm), negative ionization and full-scan and consecutive selected reaction monitoring methods with the following transitions: PC extension unit: *m/z* 287 → 125, PC terminal unit: *m/z* 289 → 245, PD extension unit: *m/z* 303 → 125 and PD terminal unit: *m/z* 305 → 125), see Engström et al. [7] for the detailed chromatographic and mass spectrometric conditions.

The mDP values were calculated according to the equation presented by Engström et al. [7] as follows:

(1) $$\mathrm{mDP}=\;\frac{0.37\times A_{287\;}+\;0.42\times A_{289}\;+\;2.15\times A_{303\;}+\;0.68\times A_{305}}{0.42\times A_{289}\;+\;0.68\times A_{305}}$$

where A_287_, A_289_, A_303_ and A_305_ are the sums of peaks areas determined from SRM chromatograms of PC extension units (*m/z* 287→ 125), PC terminal units (*m/z* 289 → 245), PD extension units (*m/z* 303 → 125) and PD terminal units (*m/z* 305 → 125), respectively, determined with the five highest cone voltages as presented in Table 2 in Engström et al. [7].

## Figures and Tables

**Table 1:** Nutrient Composition of fresh, wilted and silage of Sainfoin and Birdsfoot trefoil from the first harvest.

	DM	OM	CP	NDF	ADF
Fresh					
Sainfoin	173	915	137	398	387
Birdsfoot trefoil	161	903	204	397	306
Wilted					
Sainfoin	408	900	132	419	373
Birdsfoot trefoil	337	894	200	443	348
Silage					
Sainfoin	365	911	138	459	401
Birdsfoot trefoil	363	879	189	484	351

DM = Dry Matter (in g/kg); OM = Organic Matter (g/kg DM); CP = Crude Protein (g/kg DM); NDF = Neutral Detergent Fiber (g/kg DM); ADF = Acid Detergent Fiber (g/kg DM).
